# Real-Time ECG-Based Detection of Fatigue Driving Using Sample Entropy

**DOI:** 10.3390/e20030196

**Published:** 2018-03-15

**Authors:** Fuwang Wang, Hong Wang, Rongrong Fu

**Affiliations:** 1 School of Mechanic Engineering, Northeast Electric Power University, Jilin 132012, China; 2 School of Mechanic Engineering, Northeastern University, Shenyang 110819, China; 3 College of Electrical Engineering, Yanshan University, Qinhuangdao 066004, China

**Keywords:** HRV, sample entropy, driving fatigue, relative power spectrum ratio β/(θ + α), brain networks

## Abstract

In present work, the heart rate variability (HRV) characteristics, calculated by sample entropy (SampEn), were used to analyze the driving fatigue state at successive driving stages. Combined with the relative power spectrum ratio β/(θ + α), subjective questionnaire, and brain network parameters of electroencephalogram (EEG) signals, the relationships between the different characteristics for driving fatigue were discussed. Thus, it can conclude that the HRV characteristics (RR SampEn and R peaks SampEn), as well as the relative power spectrum ratio β/(θ + α) of the channels (C3, C4, P3, P4), the subjective questionnaire, and the brain network parameters, can effectively detect driving fatigue at various driving stages. In addition, the method for collecting ECG signals from the palm part does not need patch electrodes, is convenient, and will be practical to use in actual driving situations in the future.

## 1. Introduction

Driver fatigue is one of the major causes of fatal road accidents according to the analysis of traffic incidents causation [1,2]. Earlier research indicates that driving fatigue is responsible for 20–30% of total road fatalities [3]. Therefore, it is particularly important to accurately and rapidly detect the driving fatigue state. Researchers have investigated different methods for detecting the fatigue state, which fall into the subjective method and the objective method. The former method, which determines the driver fatigue state mainly according to drivers’ and researchers’ judgments [4,5], is easily affected by drivers’ and researchers’ artificial subjective judgment errors. Therefore, it is generally used as an auxiliary method for detecting driving fatigue, while for the latter, which mainly involves extracting and analyzing characteristics of EEG [6,7,8,9,10], electromyogram (EMG) [11], electrocardiogram (ECG) [12,13], electrooculogram (EOG) [14], visual characteristics [15], and facial movement, it is widely used in the detection of driver fatigue [16]. In recent years, researchers have been devoted to the objective method for analyzing driver fatigue.

Research shows that HRV is associated with physical fatigue [17,18]. Ltoh et al. thought it can be distinguished by a different physiological fatigue degree of the human body using the characteristics of HRV [19]. Hanlon et al. concluded that HRV changed significantly with the increase of drivers’ fatigue degree based on their driving fatigue test using motorcycle [20]. Studies have indicated that HRV is significantly reduced when the human brain becomes fatigued [21,22,23,24]. Additionally, the changes of HRV can be reflected by entropy indicators. There are different entropy methods that can be used to calculate HRV value, such as SampEn, approximate entropy (ApEn), and Kolmogorov entropy [25]. Considering the fact that fewer samples are required, we have used SampEn to analyze HRV characteristics in our study.

The EEG, which is sensitive to neural activity [26,27], is considered to be the most reliable indicator for driving fatigue state judgment [28,29]. The study has shown that the parameters (such as C and G) of the brain network change significantly when a person shifts from an alert to a fatigued state [30]. The interaction between the EEG signals from different channels has been quantified by a non-linear measure known as the Synchronization likelihood (SL) [31]. A number of classical algorithms, based on the energy [32,33] and entropy [34] for different frequency bands of EEG signals, have been used to analyze the EEG characteristics of the driver fatigue. It is pointed out that the ratio (θ + α)/β, which shows a clear indication of increasing fatigue as the ratio between the slow wave and fast wave activities increased, is a reliable indicator for detecting fatigue [35].

Although it is very accurate to judge driving fatigue state based on EEG, the EEG acquisition equipment is relatively expensive and inconvenient to carry, which causes some difficulties with regard to future popularization and application in real driving conditions. In this paper, the conductive cloth fixed on the car steering wheel is used as the ECG electrode. Compared to the conventional ECG acquisition, the method does not need the patch electrodes, which makes it easy and convenient for using in actual driving fatigue monitoring.

## 2. Materials and Methods

### 2.1. Experiment

#### 2.1.1. Subjects

The experiment was performed in simulated driving conditions. A total of 12 healthy subjects [10 males and 2 females; aged 28 ± 1.6 (S.D)], who were randomly selected from the volunteers, were arranged to participate in the experiment. All the subjects, free of medication during the experiment, were reported to have had no sleep-related disorders or history of neurological diseases and were asked to refrain from consuming any type of stimulants such as alcohol, tea, or coffee during the experiment. All subjects continuously drove for four hours (2:00 p.m.–6:00 p.m.). The process of data acquisition was divided into nine stages (stage 1—2:00 p.m., stage 2—2:30 p.m., stage 3—3:00 p.m., stage 4—3:30 p.m., stage 5—4:00 p.m., stage 6—4:30 p.m., stage 7—5:00 p.m., stage 8—5:30 p.m., stage 9—6:00 p.m.). In addition, one hour of sleep (0:30 p.m.–1:30 p.m.) was arranged for all subjects to avoid the influence of fatigue due to the lack of sleep.

#### 2.1.2. ECG

**Figure 1:**
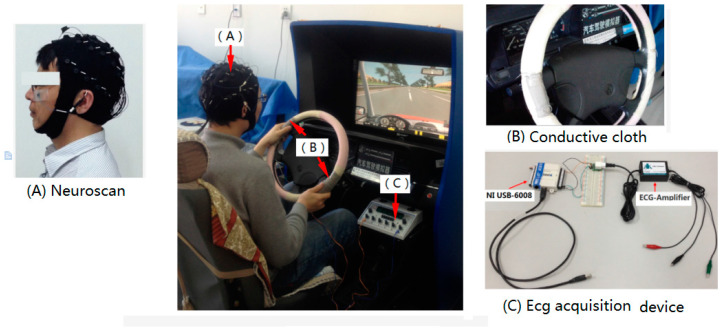
Experimental setup.

The ECG acquisition equipment in this paper mainly consists of the data acquisition card (NI USB-6008) and the signal amplifier (EKG sensor), which was used to collect the ECG signals of the palm part. The NI USB-6008 provides connection to 12 digital input/output (DIO) channels, eight analog input (AI) channels, and a 32-bit counter with a Full-Speed USB interface. The EKG sensor, which can be used to record electrical activity in the heart, can measure cardiac electrical potential waveforms. In the experiment, the ECG signal collected by the EKG sensor was transmitted to the computer serial port buffer using the NI USB-6008. Additionally, the ECG signal data were read from the serial port buffer using the LabVIEW software. Then, the driving fatigue characteristics were analyzed using the SampEn method in real time. In the process of making the equipment, we have integrated the ECG acquisition equipment and the electrical acupuncture stimulator (KWD-808I) together. Figure 1 shows the experimental set-up.

All subjects were informed about the research background and the study protocol. Additionally, they were free to choose to participate in the experiment or give up. Moreover, all of them gave their written informed consent to be included in the study. The Ethics Committee at the Northeast Electric Power University Hospital endorsed the study protocol, according to The Code of Ethics of the World Medical Association (Declaration of Helsinki).

#### 2.1.3. EEG

The Neuroscan, which is the widespread use of EEG acquisition device, is used in the experiments. Additionally, its electrodes (Ag/AgCl) are attached to the scalp according to the international 10–20 system (30 channels = FP1, FP2, F7, F3, FZ, F4, F8, FT7, FC3, FCZ, FC4, FT8, T3, C3, CZ, C4, T4, TP7, CP3, CPZ, CP4, TP8, T5, P3, PZ, P4, T6, O1, OZ, and O2). In the experiment, the EEG data recording for each stage lasts 3 min. Additionally, it should be ensured that all leads are in a normal connection state during recording data for each stage. Figure 1 shows the experimental set-up.

### 2.2. Methods

#### 2.2.1. Sample Entropy

SampEn is a modification of approximate entropy (ApEn), which is more reliable for short data sets compared with ApEn; it is used extensively for the assessment of the complexity of a physiological time-series signal. Research has shown that SampEn value reflects the stability of a system [36]. This means a system with large SampEn value approaches a random state with strong adaptability to external environment. Otherwise, a system with small SampEn value indicates a narrow spectrum band that tends to change periodically with weak adaptability. Research has shown that the SampEn value of the cardiac nervous system can reflect the regulation ability of itself [18]. In this paper, we analyzed the driver’s fatigue characteristics using the SampEn of the HRV. The SampEn is defined as follows.

Consider a time series given by ${\left\{x_{n}\right\}}_{n=1}^{N}$. Additionally, with a given embedding dimension m, the series can be denoted as

(1) $$X_{n}=\{x_{n},x_{n+1},\ldots ,x_{n+m-1}\}\in R^{m},n=N_{0},N_{0}+1,\ldots N$$

The distance function *d*[*X*(*i*), *X*(*j*)], which was used to calculate the maximum distance between *X*(*i*) and *X*(*j*), is defined as

(2) d[X(i),X(j)]=max‖x(i+k−1)−x(j+k−1)‖

in which *k* = 1, 2, …, *m*. The probability of pairs of vectors having the distance ≤ *r* is expressed as

$$C_{i}^{m}(r)=\text{\{}d[X(i),X(j)]\leq r\text{\}}/(N-m)\;i\leq N-m+1$$

in which *r* is the tolerance factor assumed for similarity between samples. Additionally, $C_{i}^{m}(r)$ needs to satisfy *i* ≠ *j* conditions. So, the SampEn is defined as

(3) $$SampEn=\ln\frac{\phi^{m}(r)}{\phi^{m+1}(r)}=\ln\frac{{\left(N-m\right)}^{-1}\sum_{i=1}^{N-m}C_{i}^{m}(r)}{{\left(N-m-1\right)}^{-1}\sum_{i=1}^{N-m}C_{i}^{m+1}(r)}$$

in which $\phi^{m}(r)=(N-m{)}^{-1}\sum_{i=1}^{N-m}C_{i}^{m}(r)$.

Based on previous studies, we calculated SampEn with the most widely-used parameter setting, i.e., *m* = 2 and *r* = 20% of the original time series standard deviation [37,38,39].

#### 2.2.2. Brain Network 

Research has shown that a number of cortical and sub-cortical regions are activated in different brain regions when human beings process complex information [40]. The rapidly changing and widely distributed neural activation will occur in brain regions during visual information processing [1]. In this paper, the data processing methods involve decomposing EEG signals into different bands using the wavelet pocket decomposition (WPD), building network using SL with a fixed threshold, and computing network parameters and other characteristics of EEG using the classical methods. The methods are explained in detail in the subsequent paragraphs.

- Preprocessing and artifact removal using WPD

In this experiment, the EEG recordings are influenced much more by noises. The noises mainly contain numerous low frequency and high frequency noises known as artifacts, such as the noises produced by the human body movement, vehicle simulator body vibration, and biological electrical signals, etc. These noises should be filtered using the useful frequency band. In this paper, the 36 Hz–44 Hz frequency band, which is associated with the human states of arousal or alertness [41,42], is extracted from the raw EEG using the WPD method.

- Formation of a brain network

The theory of modern complex networks has been used extensively to imitate human brain function [30]. Brain connectivity analysis has been proven to be a very effective and informative way to explore brain function and mental state [43,44,45]. In the paper, the structural properties of subjects’ brain networks are used to detect the changing of driving states.

**Figure 2:**
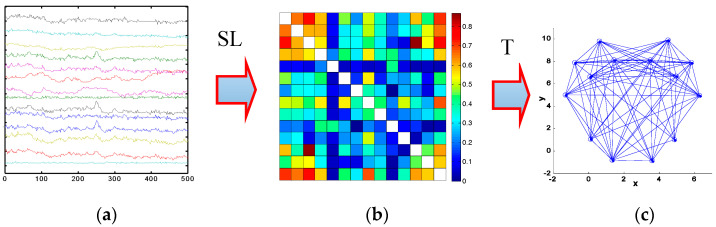
Steps of the analysis of EEG synchronization. (**a**) EEG data; (**b**) SL matrix; (**c**) Brain network.

Figure 2 shows the steps for analysis of the brain networks. In the analysis, every region of the brain is taken as a node, and the connections between brain regions are taken as edges. The steps of the brain connectivity analysis are shown in Figure 2.

In the first step, the data of a 14-channel EEG, shown in Figure 2a, are collected. Then, the sub-bands 36 Hz–44 Hz signals are extracted from original EEG signals. In the second step, the synchronization matrices, displayed in Figure 2b, are computed for the sub-bands (36 Hz–44 Hz). The correlations between pairs of 14 channels EEG are calculated using SL. The SL, which is used to describe the synchronization between 2 time series, is sensitive to nonlinear interdependencies. Additionally, its value lies in the range *P_ref_* ≤ T ≤ 1. Here, the *P_ref_* is the minimum value (a small number close to 0) in the case of independent time series, and 1 in the case of maximally synchronous signals. In this paper, Pref was set at 0.01. The result of computing the SL in this study is a square *N* × *N* matrix of size 14 (14 channels = F7, F3, F4, F8, FT7, FT8, C3, C4, TP7, TP8, P3, P4, O1, and O2), in which each entry *N_i,j_* contains the value of the SL between the channels *i* and *j*. The last step is to convert the *N* × *N* synchronization matrix into a binary graph using a threshold T. An edge is deemed to exist between *i* and *j* if the SL between a pair of channels *i* and *j* is greater than the T; otherwise, no edge exists between *i* and *j*. Finally, the networks are formed using the binary matrix with a fixed threshold value. In this study, the degree of connectivity, the cluster coefficient C and global efficiency, and the main structural properties parameters of a network are used to analyze the functional differences of the complex brain networks. These are explained here.

- Degree of connectivity (Ki)

The connectivity degree of a node indicates the importance of that node in a network, which can be represented as the number of edges connected to that node.

1. Clustering Coefficient 

The cluster coefficient *C* is a measure of the local structure of network, which can be expressed as the ratio of the number of existing edges and the number of maximum possible edges between neighbors of a node [46,47]. Its formula can be defined as:

(4) $$C_{i}=\frac{e_{i}}{k_{i}(k_{i}-1)/2}$$

in which *e_i_* is the number of existing edges between neighbors of the node *i.* Additionally, *k_i_* is the degree of connectivity of that node. *k_i_*(*k_i_* − 1)/2 is the number of maximum possible edges between neighbors of the node *i* [32]. Mean cluster coefficient *C* of the graph is represented as [30].

(5) $$C=\frac{1}{N_{e}}\sum_{i=1}^{N_{e}}C_{i}$$

in which *N_e_* is the total number of nodes or electrodes.

2. Global efficiency

The global efficiency is a global structural characteristic of a network, which indicates that the higher level the integration of a network, the faster the information transfer. The path length between two nodes *i* and *j*, *L_i,j_*, is the minimum number of edges that are needed to connect. The characteristic path length, which connects a particular node *i* with the rest of the network, is the mean of *L_i,j_* over the entire network. Moreover, the path length is inverse ratio with the nodal efficiency is mathematically defined as [32,48]:

(6) $$E_{nodal(i)}=\frac{1}{N-1}\sum_{j\in G}\frac{1}{L_{i,j}}$$

in which *L_i,j_* is the minimum path length (the smallest number of intervening edges) between nodes *i* and *j*. Additionally, *N* is the number of nodes within the graph. The average value of the nodal efficiencies of each node can be used to estimate the global efficiency *G*. So, the global efficiency of nodes can be defined by:

(7) $$G=E_{global}=\frac{1}{N(N-1)}\sum_{i\neq j\in G}\frac{1}{L_{i,j}}$$

From the Equation (7), one can see that networks, which have a highly integrated organization and are characterized by short minimum path length between any pair of regional nodes, have high global efficiency [49,50]. Combined with the Equation (5), this leads to the fact that the smaller *L_i_*, *j*, the faster information transmission speed of a node with others. 

#### 2.2.3. The Relative Power Spectrum

Four frequency sub-bands (δ (0–4 Hz), θ (4–8 Hz), α (8–13 Hz), and β (13–35 Hz)) are widely applied to analyze the state of driving fatigue. Their power spectrum ratios have different combinations, such as θ/β, θ/α + β, θ + α/β, θ + α/α + β, β/α, which can show different characteristics of driving fatigue over time [32,51,52]. Significant changes, compared with the algorithms θ/β, α/β and (θ + α)/(α + β), were found for the algorithm (θ + α)/β at the monotonous driving sessions [32,51]. In addition, the brain fatigue characteristics can be easily detected from the frontal (F3, F4), central (C3, C4), and posterior (P3, P4) brain regions using EEG signals [35]. Therefore, the leads associated with the brain regions can be used as the preferred ones for the analysis of driving fatigue in this study. From what has been discussed above, the relative power spectrum ratio (θ + α/β) is used to analyze driver fatigue using the EEG signals collected from the channels F3, F4, P3, and P4. In view of the data variation characteristics of brain networks, the ratio (θ + α)/β is denoted as β/(θ + α) for convenient comparison with the parameter SampEn value.

#### 2.2.4. Statistical Analysis Algorithm

In order to compare the differences of detection results, the statistical analysis methods (ANOVA and Tukey test) were used in this study. In the comparative analysis section, ANOVA were used to compare the proposed method (Sample entropy) with compared methods (brain network, relative power spectrum, and subjective questionnaire) and follow a multiple comparisons test that we gave using Tukey test to identify significant differences between the driving fatigue state with other driving states.

## 3. Results

### 3.1. HRV Characteristics

**Figure 3:**
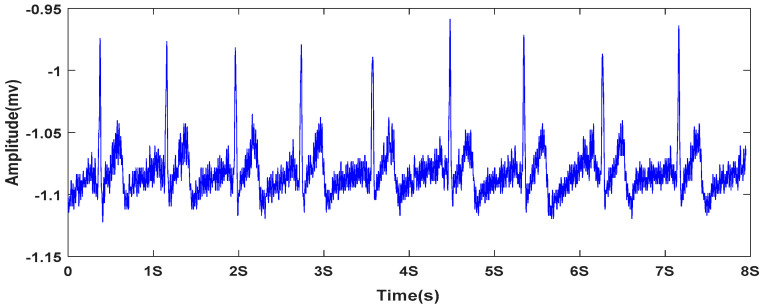
The ECG of one subject.

Research shows that the HRV is associated with physical fatigue [17,18,19]. In this paper, we used the SampEn of the HRV characteristics (R-Peaks series and RR intervals series) to analyze the changes of driving fatigue. Figure 3 shows the ECG signal that was acquired from the palm of one of the subject hands.

**Figure 4:**
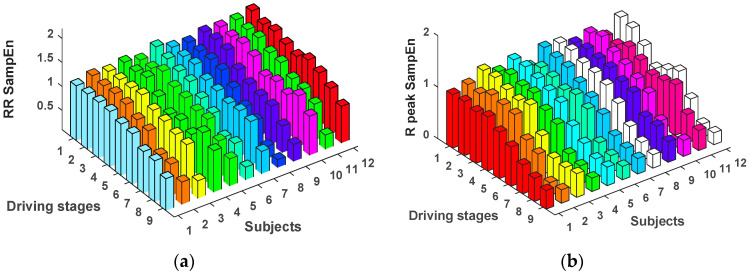
Tendency changes of SampEn values in 9 experimental stages. (**a**) RR SampEn; (**b**) R-Peak SampEn.

The analysis of HRV characteristics was carried out for 12 subjects in 9 stages (stages 1–9). Additionally, the SampEn values were calculated separately for RR intervals series and R-Peaks series. Throughout the experimental phase, the tendency changes of SampEn values in 9 experimental stages are shown in Figure 4.

Figure 4 shows that the SampEn values of RR intervals series and R-Peaks series present general downward trend in the process of the whole experiment. The two types of SampEn values have a slight decline from stage 1 to 5. This means the subjects began to get a little tired. However, a significant decline, which occurs at the experimental stage 5 to 7, means the subjects’ degree of driving fatigue increases gradually.

### 3.2. Brain Network

#### 3.2.1. Choice Threshold T

**Figure 5:**
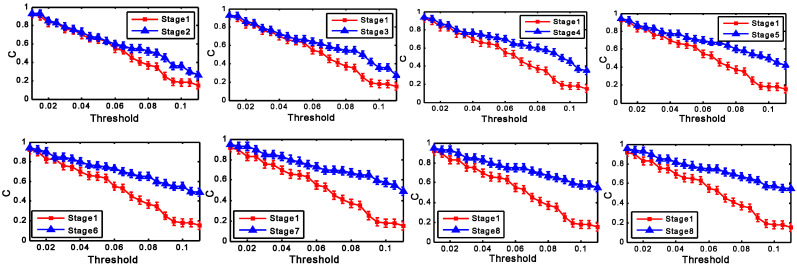
Mean cluster coefficient C as a function of threshold for different groups.

To compare the cluster coefficient C of brain networks at different stages, the networks have been formed at all the thresholds for each stage. In general, the choice of threshold should depend on the research question and falls in the regime of educated guesses [53]. In present work, we explored a whole range of values of T, 0.01 < T < 0.11, with increments of 0.005, and repeated the full calculation for each value of T. Figure 5 shows the comparation of the cluster coefficient C at different stages.

Figure 5 shows the differences of the mean cluster coefficient C for different groups with the changes of the threshold T. The changes of C in all stages present a downward trend. This is because more and more edges will be lost with the increasing values of the threshold. Over the whole range of threshold values (0.01–0.11), the significant difference of C can be found for different groups in the case that T is chosen in the range 0.06 < T < 0.11. With the same method, the significant difference of G can be found for different groups in the case that T is chosen in the range 0.08 < T < 0.11. The mean value of T can be calculated when T lies in the range 0.08 < T < 0.11. In present study, the mean value of T (T = 0.095) is chosen as the fixed threshold. With the fixed threshold, the network parameters C and G for all the subjects at different stages have been computed.

#### 3.2.2. Cluster Coefficient C and Global Efficiency G

**Figure 6:**
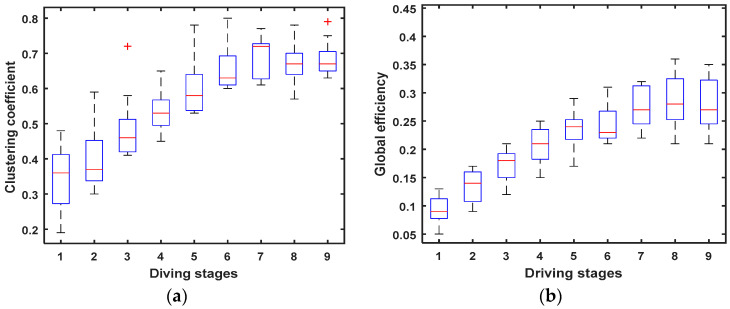
Variation tendency of cluster coefficient C and global efficiency G at 9 driving stages. (**a**) Cluster coefficient of the brain networks; (**b**) Global efficiency of the brain networks.

The cluster coefficient C and global efficiency G of the brain network are calculated, respectively, using the Equations (3) and (5), and their variation tendency at 9 driving stages is shown in Figure 6. 

Figure 6 shows that the increase in cluster coefficient C and global efficiency G can be significantly observed at successive driving stages in 36 Hz–44 Hz sub-band. Research shows that the upward changes of the two parameters (C and G) of the brain networks indicated a lack of alertness [39,41]. So, the cluster coefficient C and global efficiency G, shown in Figure 6, demonstrate an increased synchronisation between EEG signals from different brain regions in the sub-band 36 Hz–44 Hz with the driving fatigue increasing at successive stages. Thus, we can conclude that the changes in the above parameters can effectively reflect higher fatigue levels at successive stages in the experiment.

### 3.3. The Relative Power Spectrum

**Figure 7:**
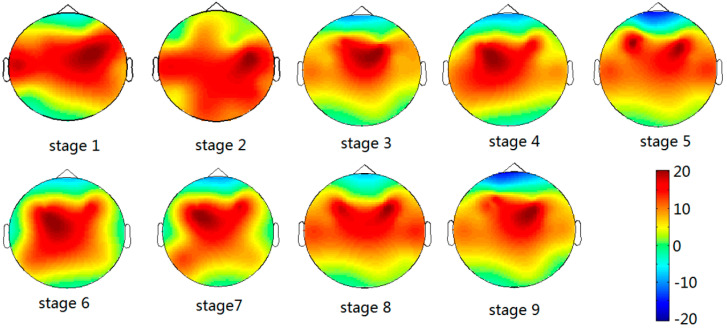
Brain topography of one subject in 9 driving stages.

The power spectrum is a commonly used parameter in the analysis of driving fatigue. Figure 7 shows the brain topography, which indicates nerve activities for one of the subjects. For brain topography, low activity is indicated by the blue-shaded areas, whereas high activity is indicated by the red-shaded areas. Figure 7 shows that the brain activity decreased steadily in the brain regions (C3, C4, P3, and P4) in driving stages 1 to 9.

**Figure 8:**
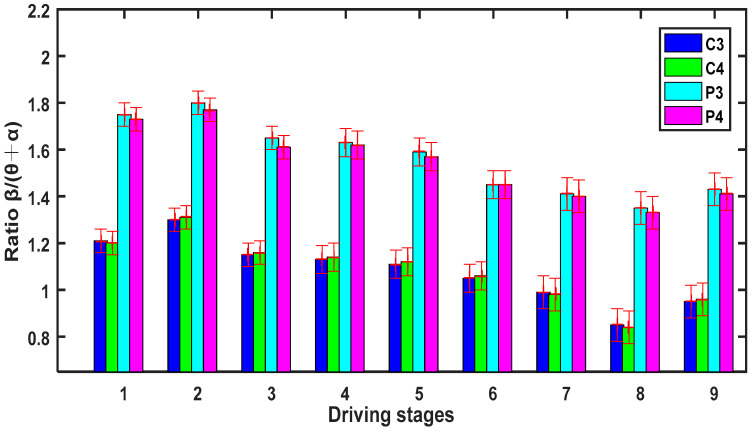
Ratio β/(θ + α) (mean ± s.d.) of the relative power spectrum for 9 stages of driving.

Figure 8 shows that the ratio β/(θ + α) of the relative power spectrum for the four channels (C3, C4, P3, and P3) presents a downward trend at successive stages (*p* < 0.05), and that means the degrees of brain activity suppressed by inhibition are growing and the fatigue degrees of drivers are deepening gradually at increasing stages of the experiment.

### 3.4. Subjective Questionnaire

**Figure 9:**
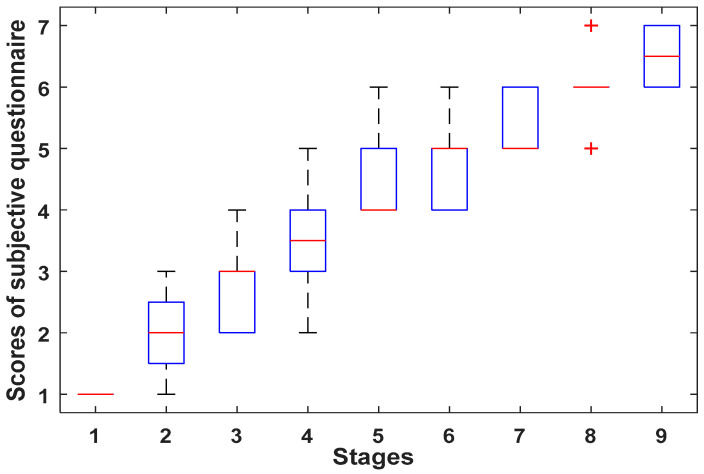
Scores (mean ± s.d.) of subjective questionnaire for 9 stages of driving.

Research has shown subjective questionnaire (SQ) is a common way to detect human fatigue [54,55]. In this paper, we used the 7-point Samne Perelli Fatigue Scale (1—Fully alert, wide awake, 2—Very lively, responsive, but not at peak, 3—Okay, somewhat fresh, 4—A little tired, less than fresh, 5—Moderately tired, let down, 6—Extremely tired, very difficult to concentrate, 7—Completely exhausted, unable to function effectively) to judge driving fatigue level. At each stage of the experiment, participants were asked about their subjective fatigue and give a score. The scores of subjective questionnaire are shown in Figure 9.

Figure 9 shows that the scores of subjective questionnaire for 9 stages of driving present an upward trend at successive stages, and that means that the subjective fatigue degrees of subjects are deepening gradually at increasing stages of the experiment. 

### 3.5. Comparative Analysis 

#### 3.5.1. Correlation Analysis

The comparative analysis involves the relationship between the pairs of parameters (RR SampEn, R peaks SampEn, C, G, F3_β/θ+α_, P4_β/θ+α_, C3_β/θ+α_, C4_β/θ+α_, SQ). In order to investigate correlations between changes in drivers fatigue with brain activities, we calculated Pearson’s correlation coefficient between pairs of the parameters. The correlation coefficients between RR SampEn, R peaks SampEn, C, G, F3_β/θ+α_, P4_β/θ+α_, C3_β/θ+α_, C4_β/θ+α_, and SQ were calculated. The results are shown in Table 1. 

The Table 1 shows that the absolute values of correlation coefficients between pairs of variables (RR SampEn, R peaks SampEn, C, G, F3_β/θ+α_, P4_β/θ+α_, C3_β/θ+α_, C4_β/θ+α_, SQ) are greater than 0.50. It means that these variables have a strong correlation. Especially, the correlation coefficients between the HRV parameter (RR SampEn and R peaks SampEn) and EEG parameters, which are greater than 0.65, mean there is a stronger correlation between them. Thus, we can conclude that the HRV characteristics (RR SampEn and R peaks SampEn), as well as the relative power spectrum ratio β/(θ + α) of the channels (C3, C4, P3, P4), subjective questionnaire, and the brain network parameters, can detect driver fatigue at various stages of the experiment. 

#### 3.5.2. HRV Characteristics and Subjective Questionnaire

The 7-point Samne Perelli Fatigue Scale (1—Fully alert, wide awake, 2—Very lively, responsive, but not at peak, 3—Okay, somewhat fresh, 4—A little tired, less than fresh, 5—Moderately tired, let down, 6—Extremely tired, very difficult to concentrate, 7—Completely exhausted, and unable to function effectively) divides subjective fatigue into different fatigue grades. Among these fatigue grades, “Moderately tired, let down” state can make a person’s reaction become slow, even causing misoperation, which is incompatible with continued driving. So, accurately detecting this fatigue state is of great significance for improving safe driving. In our study, the SampEn of the HRV characteristics (R-Peaks series and RR intervals series) were used to analyze the changes in driving fatigue. The comparison of significant differences between the different subjective fatigue states was analyzed using the statistical analysis methods (ANOVA and Tukey test). The results are shown in Table 2.

Table 2 shows that there are significant differences between “Moderately tired, let down” state and other different subjective fatigue states (*p* < 0.05). This means that the subjective fatigue state “Moderately tired, let down” can be distinguished using the HRV characteristics (R-Peaks series and RR intervals series). The sample entropy values of main subjective fatigue stages (Fully alert, wide awake; A little tired, less than fresh; Moderately tired, let down; Extremely tired, very difficult to concentrate) are shown in Table 3. 

Table 3 shows the SampEn values of the HRV characteristics in main subjective fatigue stages. Additionally, the SampEn values of the two subjective fatigue states (A little tired, less than fresh and Moderately tired, let down) are obviously different. So, we can conclude that a driver is in a state of driving fatigue when the SampEn values of his HRV characteristics satisfy the condition (RR SampEn < 0.8053 and R-Peak SampEn < 0.7258).

#### 3.5.3. Methods Comparison

In our study, several typical traditional detection methods, which can effectively detect driving fatigue, were introduced to verify the effectiveness of the method (the SampEn) for driving fatigue analysis. Additionally, the statistical analysis methods (ANOVA and Tukey test) were used to analyze the differences of fatigue states for different methods. Then, the identification effect of these methods was calculated. The results are shown in Table 4.

Table 4 shows that there are significant differences (*p* < 0.05) between “Moderately tired, let down” state with other different subjective fatigue states when we analyze driving fatigue using each method. Comparison between the three methods and the identification effect of the method based on brain network are most significant. Although the recognition effect of the SampEn method is not the best, it can also detect significant differences between driving fatigue state and other fatigue grade states.

## 4. Discussion

Driver fatigue is more likely to bring serious safety trouble to traffic. Much research has been carried out on driving fatigue [6,7,8,9,10,11,12,13,14,15]. Research has shown that the method based on EEG, which is sensitive to neural activity [26,27], is considered to be the most reliable indicator for driving fatigue state judgment [28,29]. In our study, the validity of the HRV method was proved by using these EEG parameters as references. 

In our experiment, the subjects need to have more than one year driving experience. Additionally, the twelve subjects were randomly selected from the volunteers. Additionally, the EEG data of subjects were analyzed. The results showed that the EEG characteristic parameters showed regular changes (Figure 6 and Figure 8), which were consistent with previous studies [32,51,56,57]. Therefore, we can try to use these results as the basis of judging the correlation between HRV characteristics and driving fatigue. The final result (Table 1, Table 2 and Table 4,) showed that one can effectively distinguish between driving fatigue state and other fatigue grade states using the HRV characteristics. 

In addition, the ratio of fatal accidents involving male and female drivers, according to a survey conducted by the Jiangsu Public Security Department, was about 9 to 1 in 2014 [58]. Additionally, research has indicated that driving fatigue is responsible for 20–30% of total road fatalities [3]. So, in our experiment, the subjects included 10 males and 2 females.

### 4.1. Previous Studies

Research has shown that the EEG, which is sensitive to neural activity, is the most reliable indicator for driving fatigue state judgment [8,9,27,28,29]. Additionally, the subjective questionnaire is an effective method for detecting human fatigue [54,55]. Although it is very accurate to judge driving fatigue state based on EEG, the EEG acquisition devices are relatively expensive and inconvenient to carry, which brings some difficulties to the future popularization and application of real driving conditions. Additionally, the subjective questionnaire method may not be effectively performed by drivers in actual driving.

### 4.2. Novel Findings of This Study

Our results showed that the SampEn method could identify driving fatigue as effectively as the conventional methods (brain Network, relative power spectrum, and Subjective questionnaire). Additionally, this ECG acquisition equipment, which has a lower price than the traditional EEG equipment, is convenient and practical for use in actual driving. In addition, in our experiment, the ECG data were collected and analyzed online, which was of great significance for future practical application.

### 4.3. Limitations and Future Research Lines

In our experiment, subjective questionnaire was used to detect human fatigue. The experiment was divided into nine time periods. Each time period lasted for 30 min. Additionally, the subjective questionnaire was filled out at the end of each time period. It is possible that the subjective fatigue state changed during the time period, which might have caused some small errors in the statistical results. In future research works, portable equipment that can accurately and rapidly detect driving fatigue state and alleviate fatigue will be developed.

## 5. Conclusions

The major finding of the above study is that the ECG signals collected from the palm part can effectively detect drivers’ fatigue. In the experiment, the results from the network analysis suggest an increase in the degree of connectivity at increased levels of driving fatigue. Further, the decreasing trend in the relative power spectrum ratio β/(θ + α) suggests the increasing effects of driving fatigue at successive driving stages. Finally, the correlation study between pairs of variables (RR SampEn, R peaks SampEn, C, G, F3_β/θ+α_, P4_β/θ+α_, C3_β/θ+α_, C4_β/θ+α_ SQ) allows one to draw the conclusion that these variables have a strong correlation, especially with regard to the correlation coefficients between the HRV parameter (RR SampEn and R peaks SampEn) and the EEG parameters. Therefore, it can be concluded that the ECG signals collected from the palm part can effectively monitor driving fatigue, which is helpful for improving safe driving for long time driving. In addition, this method needs not patch electrodes, is convenient, and is practical for use in actual driving situations in the future.

## Figures and Tables

**Table 1:** Correlation coefficient.

	SampEn (RR)	SampEn (R Peaks)	C	G	P3_β/θ+α_	P4_β/θ+α_	C3_β/θ+α_	C4_β/θ+α_	SQ
SampEn (RR)	1	0.8578	−0.6637	−0.7133	0.8035	0.8167	0.7651	0.7411	−0.9531
SampEn (R peaks)	0.8578	1	−0.6781	−0.7103	0.7792	0.7619	0.7366	0.7098	−0.8909
C	−0.6637	−0.6781	1	0.9576	−0.8356	−0.7960	−0.6513	−0.6845	0.7764
G	−0.7133	−0.7103	0.9576	1	−0.8501	−0.8278	−0.6812	−0.6988	0.7452
P3_β/θ+α_	0.8035	0.7792	0.8356	−0.8501	1	0.9822	0.8834	0.8602	−0.8055
P4_β/θ+α_	0.8167	0.7619	0.7960	−0.8278	0.9822	1	0.8577	0.8425	−0.8134
C3_β/θ+α_	0.7651	0.7366	−0.6513	−0.6812	0.8834	0.8577	1	0.8919	−0.7531
C4_β/θ+α_	0.7411	0.7098	−0.6845	−0.6988	0.8602	0.8425	0.8919	1	−0.7319
SQ	−0.9531	−0.8909	0.7764	0.7452	−0.8055	−0.8134	−0.7531	−0.7319	1

**Table 2:** The compardison of significant differences between “Moderately tired, let down” state with other different subjective fatigue states using the sample entropy.

Subjects	Probability Value	Stage 1	Stage 2	Stage 3	Stage 4	Stage 5	Stage 6	Stage 7	Stage 8	Stage 9
Subject 1	P_RR Sampan _(Fatigue Scale)	2.6686 × 10^−4^ (1)	0.0018 (2)	0.0023 (3)	0.0396 (4)	0.0396 (4)	1 (5)	1 (5)	0.0137 (6)	0.0043 (7)
	P_R-Peak SampEn_ (Fatigue Scale)	2.9266 × 10^−4^ (1)	0.0033 (2)	0.0059 (3)	0.0443 (4)	0.0443 (4)	1 (5)	1 (5)	0.0261 (6)	0.0078 (7)
Subject 2	P_RR SampEn_ (Fatigue Scale)	2.3667 × 10^−4^ (1)	2.3667 × 10^−4^ (1)	0.0019 (2)	0.0019 (2)	0.0165 (4)	1 (5)	1 (5)	0.0217 (6)	0.0217 (6)
	P_R-Peak SampEn_ (Fatigue Scale)	2.8263 × 10^−4^ (1)	2.8263 × 10^−4^ (1)	0.0023 (2)	0.0023 (2)	0.0122 (4)	1 (5)	1 (5)	0.0115 (6)	0.0115 (6)
Subject 3	P_RR SampEn_ (Fatigue Scale)	6.2561 × 10^−5^ (1)	6.2561 × 10^−5^ (1)	0.0013 (2)	0.0185 (3)	0.0449 (4)	0.0449 (4)	1 (5)	0.0377 (6)	0.0377 (6)
	P_R-Peak SampEn_ (Fatigue Scale)	8.8713 × 10^−5^ (1)	8.8713 × 10^−5^ (1)	0.0077 (2)	0.0238 (3)	0.0316 (4)	0.0316 (4)	1 (5)	0.0192 (6)	0.0192 (6)
Subject 4	P_RR SampEn_ (Fatigue Scale)	2.5912 × 10^−5^ (1)	0.0011 (2)	0.0115 (3)	0.0115 (3)	0.0399 (4)	0.0399 (4)	1 (5)	0.0296 (6)	0.0296 (6)
	P_R-Peak SampEn_ (Fatigue Scale)	3.1764 × 10^−5^ (1)	0.0026 (2)	0.0188 (3)	0.0188 (3)	0.0471 (4)	0.0471 (4)	1 (5)	0.0366 (6)	0.0366 (6)
Subject 5	P_RR SampEn_ (Fatigue Scale)	4.5612 × 10^−4^ (1)	0.0025 (2)	0.0246 (3)	0.0483 (4)	1 (5)	1 (5)	0.0419 (6)	0.0419 (6)	0.0419 (6)
	P_R-Peak SampEn_ (Fatigue Scale)	3.9371 × 10^−4^ (1)	0.0012 (2)	0.0211 (3)	0.0392 (4)	1 (5)	1 (5)	0.0388 (6)	0.0388 (6)	0.0388 (6)
Subject 6	P_RR SampEn_ (Fatigue Scale)	6.2613 × 10^−5^ (1)	0.0017 (2)	0.0017 (2)	0.0093 (3)	0.0274	0.0274	1 (5)	1 (5)	0.0436 (6)
	P_R-Peak SampEn_ (Fatigue Scale)	3.5732 × 10^−5^ (1)	0.0012 (2)	0.0012 (2)	0.0037 (3)	0.0127	0.0127	1 (5)	1 (5)	0.0335 (6)
Subject 7	P_RR SampEn_ (Fatigue Scale)	0.0044 (1)	0.0199 (3)	0.0478 (4)	1 (5)	0.0226 (6)	0.0226 (6)	0.0226 (6)	0.0031 (7)	0.0031 (7)
	P_R-Peak SampEn_ (Fatigue Scale)	0.0013 (1)	0.0175 (3)	0.0417 (4)	1 (5)	0.0352 (6)	0.0352 (6)	0.0352 (6)	0.0018 (7)	0.0018 (7)
Subject 8	P_RR SampEn_ (Fatigue Scale)	0.0016 (1)	0.0215 (2)	0.0386 (4)	0.0386 (4)	1 (5)	1 (5)	0.0483 (6)	0.0483 (6)	0.0162 (7)
	P_R-Peak SampEn_ (Fatigue Scale)	0.0011	0.0224 (2)	0.0414 (4)	0.0414 (4)	1 (5)	1 (5)	0.0446 (6)	0.0446 (6)	0.0126 (7)
Subject 9	P_RR SampEn_ (Fatigue Scale)	0.0025 (1)	0.0178 (3)	0.0178 (3)	0.0415 (4)	0.0415 (4)	1 (5)	0.0361 (6)	0.0361 (6)	0.0021 (7)
	P_R-Peak SampEn_ (Fatigue Scale)	0.0031 (1)	0.0199 (3)	0.0199 (3)	0.0471 (4)	0.0471 (4)	1 (5)	0.0427 (6)	0.0427 (6)	0.0036 (7)
Subject 10	P_RR SampEn_ (Fatigue Scale)	3.4407 × 10^−4^ (1)	0.0037 (2)	0.0131 (3)	0.0435 (4)	0.0435 (4)	1 (5)	0.0481 (6)	0.0481 (6)	0.0065 (7)
	P_R-Peak SampEn_ (Fatigue Scale)	4.1711 × 10^−4^ (1)	0.0017 (2)	0.0061 (3)	0.0355 (4)	0.0355 (4)	1 (5)	0.0412 (6)	0.0412 (6)	0.0033 (7)
Subject 11	P_RR SampEn_ (Fatigue Scale)	5.6702 × 10^−4^ (1)	5.6702 × 10^−4^ (1)	0.0027 (2)	0.0087 (3)	0.0279 (4)	0.0279 (4)	1 (5)	1 (5)	0.0396 (6)
	P_R-Peak SampEn_ (Fatigue Scale)	9.0146 × 10^−4^ (1)	9.0146 × 10^−4^ (1)	0.0046 (2)	0.0112 (3)	0.0318 (4)	0.0318 (4)	1 (5)	1 (5)	0.0413 (6)
Subject 12	P_RR SampEn_ (Fatigue Scale)	0.0016 (1)	0.0078 (2)	0.0078 (2)	0.0466 (3)	1 (5)	1 (5)	1 (5)	0.0413 (6)	0.0413 (6)
	P_R-Peak SampEn_ (Fatigue Scale)	7.1642 × 10^−4^ (1)	0.0054 (2)	0.0054 (2)	0.0419 (3)	1 (5)	1 (5)	1 (5)	0.0359 (6)	0.0359 (6)

The 7-point Samne Perelli Fatigue Scale: (1) Fully alert, wide awake; (2) Very lively, responsive, but not at peak; (3) Okay, somewhat fresh; (4) A little tired, less than fresh; (5) Moderately tired, let down; (6) Extremely tired, very difficult to concentrate; (7) Completely exhausted, unable to function effectively.

**Table 3:** The SampEn values (mean ± S.D.) of main subjective fatigue stages.

	Fully Alert, Wide Awake	A Little Tired, Less Than Fresh	Moderately Tired, Let Down	Extremely Tired, Very Difficult to Concentrate
RR SampEn	1.3081 ± 0.0965	1.1575 ± 0.0615	0.8053 ± 0.0833	0.5419 ± 0.1059
R-Peak SampEn	1.1967 ± 0.0792	1.0813 ± 0.0922	0.7258 ± 0.0943	0.3255 ± 0.1127

**Table 4:** The comparison of significant differences between “Moderately tired, let down” state with other different subjective fatigue states for different methods.

	Methods	Fully Alert, Wide Awake	A Little Tired, Less Than Fresh	Moderately Tired, Let Down	Extremely Tired, Very Difficult to Concentrate
Moderately tired, let down	SampEn (RR)	0.0071	0.0437	1	0.0245
	SampEn (R peaks)	0.0104	0.0453	1	0.0336
	C	0.0043	0.0153	1	0.0246
	G	0.0182	0.0287	1	0.0215
	P3_β/θ+α_	0.0082	0.0359	1	0.0288
	P4_β/θ+α_	0.0057	0.0361	1	0.0291
	C3_β/θ+α_	0.0106	0.0375	1	0.0372
	C4_β/θ+α_	0.0113	0.0419	1	0.0355
